# The Role of Mammographic Calcification in the Neoadjuvant Therapy of Breast Cancer Imaging Evaluation

**DOI:** 10.1371/journal.pone.0088853

**Published:** 2014-02-11

**Authors:** Jun-jie Li, Canming Chen, Yajia Gu, Genhong Di, Jiong Wu, Guangyu Liu, ZhiMin Shao

**Affiliations:** 1 Department of Breast Surgery, Cancer Center and Cancer Institute, Shanghai Medical College, Fudan University, Shanghai, China; 2 Department of Diagnostic Radiology, Cancer Center and Cancer Institute, Shanghai Medical College, Fudan University, Shanghai, China; Taipei Medicine University, Taiwan

## Abstract

**Introduction:**

Investigate the patterns of mammographically detected calcifications before and after neoadjuvant chemotherapy (NACT) to determine their value for efficacy evaluation and surgical decision making.

**Methods:**

187 patients with malignant mammographic calcifications were followed to record the appearances and changes in the calcifications and to analyze their responses to NACT.

**Results:**

Patients with calcifications had higher rates of hormonal receptor (HR) positive tumors (74.3% versus 64.6%) and HER2 positive tumors (51.3% versus 33.4%, p = 0.004) and a similar pathologic complete response (pCR) rate compared to patients without calcifications (35.4% versus 29.8%). After NACT, the range of calcification decreased in 40% of patients, increased in 7.5% and remained stable in 52.5%; the calcification density decreased in 15% of patients, increased in 7.5% and remained stable in 77.5%; none of these change patterns were related to tumor response rate. No significant correlation was observed between the calcification appearance (morphology, distribution, range, diameter or density) and tumor subtypes or pCR rates. Among patients with malignant calcifications, 54 showed calcifications alone, 40 occurred with an architectural distortion (AD) and 93 with a mass. Calcifications were observed inside the tumor in 44% of patients and outside in 56%, with similar pCR rates and patterns of change.

**Conclusions:**

Calcification appearance did not clearly change after NACT, and calcification patterns were not related to pCR rate, suggesting that mammogram may not accurate to evaluate tumor response changes. Microcalcifications visible after NACT is essential for determining the extent of excision, patients with calcifications that occurred outside of the mass still had the opportunity for breast conservation.

## Introduction

Neoadjuvant chemotherapy (NACT) is the preferred approach for patients with locally advanced breast cancer (LABC) [1], [2]. Historically, NACT was used to induce tumor shrinkage and to improve the disease-free survival in patients with LABC considered inoperable at diagnosis [3], [4]. Several randomized trials have demonstrated that NACT may improve the resectability rate, offering disease-free and overall survival rates that are at least equivalent to those offered by surgery alone [5]–[7]and a statistically significant increase in the use of breast conserving therapy (BCT) over mastectomy [8], [9]. Precise measurement of responses to NACT is essential for surgical decision making, includes physical examination, mammogram (MG), sonography and MRI [10]. The standard recommended assessment method is to repeat these image evaluations before and after neoadjuvant.

MG is the most commonly used diagnostic imaging modality to estimate primary tumor range at the time of diagnosis, and its implementation has contributed to an increase in early diagnosis and a decline in breast cancer mortality [11]. Referring to the fourth Breast Imaging Reporting and Data System (BIRADS) categorization[12], the most typical and distinctive features on MG are microcalcifications, which suggest malignant breast cancer. Many studies have compared MG, sonography and MRI in patients undergoing NACT [13]–[24]. The results indicated that MG and sonography are accurate in measuring tumor size at the time of diagnosis, but they have limited sensitivity to the residual tumor, preventing accurate assessment of the pathological response. MRI has been demonstrated to be the most reliable technique for evaluating residual disease after NACT [12], [17]–[19], [22], [23].

In cases where the cancer is associated with microcalcifications, the use of MG may be important because calcifications are not reliably identified on MRI. However, the variable appearance and the patterns of change in calcifications observed by MG have not been thoroughly assessed. Considering the current limited amount of data about the predictive value of MG calcifications in NACT, we analyzed 187 NACT patients with malignant calcifications. The purposes of this study were to document the calcification appearance and pattern of change before and after NACT, to correlate these observations with the pathological changes observed in the surgical specimens and to evaluate their influences on surgical options.

## Patients and Methods

### Study subjects

The study subjects were selected from the Breast Malignancy Database established by the Department of Surgery, Shanghai Cancer Center of Fudan University in Shanghai, China. Patients with histological confirmation by core needle biopsy (CNB) of large operable or locally advanced breast cancer without prior treatment were considered eligible for a phase II prospective NACT trial conducted by our institution. The protocol was reviewed and approved by the Ethical Committee of Shanghai Cancer Center, and all patients provided written informed consent before inclusion in this trial. The results of this trial have been previously reported [24]. All eligible patients received 4 cycles of weekly paclitaxel plus carboplatin. Herceptin was also administered to Her2 positive patients. A diagnostic CNB was allowed to establish estrogen receptor (ER), progesterone receptor (PR), HER2 and Ki67 status. Meanwhile, patients were routinely assessed by MG, ultrasound and breast MRI, bone scan and chest CT scan prior to therapy. After chemotherapy, MG, ultrasound and breast MRI and chest CT were repeated to evaluate the patient response. Patients with operable diseases after chemotherapy should receive surgery within 4 weeks from the last scheduled cycle of chemotherapy.

The trial enrolled 732 patients between 2008.2 and 2013.3, of which 187 patients with MG calcifications with a malignant appearance were eligible for assessment. The control group consisted of 48 patients (randomly selected in the same period with detail records) without malignant calcifications in MG.

### Mammography evaluation

MGs were completed using a standard four view film/screen MG. All of the digital imaging results were preserved in the PACS image system database. Pre- and post-chemotherapy MGs were assessed retrospectively, both in CC and MLO images. If an MG image indicated malignant calcifications, we conducted a detailed image analysis to assess the following features of the calcifications: morphology, distribution, range, diameter and density. One radiologist and one surgical oncologist retrospectively evaluated all the MGs, and one senior radiologist reviewed in consensus. Calcifications were classified according to the BI-RADS classification system [12]: calcification morphology was divided into fine branching or casting, pleomorphic or combined; the distribution was classified as grouped or clustered, linear, segmental, regional, diffuse or scattered; In this study, we also performed some other measurements such as range, diameter and density to more comprehensively assess the appearance of these calcifications. The range was divided into ≤2 cm or >2 cm; the diameter was divided into 3 groups of ≤0.5 mm, ≤1 mm and >1 mm; and the density was calculated as the mean number per cm^2^, grouped into ≤20 or >20.

### Immunohistochemical (IHC) evaluation and subtype classification

The main biomarkers ER, PR and Her2 were assessed by IHC in paraffin-embedded tumor samples before treatment. The cutoff for ER positivity (ER+) and PR positivity (PR+) was 1% positive tumor cells with nuclear staining. The results of Her2 3+ by IHC or positive on FISH were considered Her2 positive (Her2+), whereas cases with 0 to 1+ or 2+ without FISH detection were regarded as negative (Her2−). Hormonal receptor (HR) positive was defined as either ER+ or PR+, and HR- was defined as both ER- and PR-. Patients were categorized based on the IHC HR and Her2 status of their primary tumors. Therefore, four breast cancer subtypes were classified as follows: HR+ (HR+/Her2−), triple positive (HR+/Her2+), triple negative (HR−/Her2−) and Her2 positive (HR−/Her2+).

### Response evaluation

pCR was defined as the absence of any residual invasive cancer upon hematoxylin and eosin evaluation of the resected breast specimen. Residual ductal carcinoma in situ (DCIS) was included in the pCR category [25]. Total pCR (tpCR) was defined as the absence of any tumor (invasive carcinoma and CIS) in the final surgical breast sample.

### Statistics

Associations between categorical variables were tested using Pearson's x2 test. Logistic regression analysis was performed to determine the ability of calcification appearance to predict the tumor response rate. All statistical tests were two-sided and carried out at a significance level of 0.05 using the SPSS statistical software package (version 15.0; SPSS Company, Chicago, IL).

## Results

### Patient characteristics

**Figure 1:**
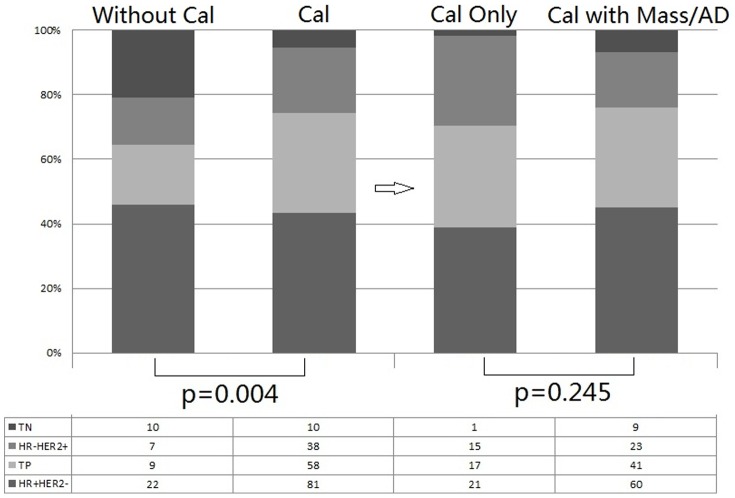
Distribution of histological subtypes according to MG appearances. Cal: calcification; AD: architectural distortion; TN: triple negative; TP: triple positive. * Histological subtypes were achieved by core needle biopsy and MG before NACT were evaluated for calcification appearance.

Overall, 732 consecutive patients were enrolled in this NACT trial. A total of 187 patients with MG calcifications with malignant appearances were eligible for assessment, accounting for one-quarter of all patients. Among this group, 6 patients did not have clear surgical records and 61 missed the MG after NACT. The control group consisted of 48 patients without calcifications with a malignant appearance. Among 187 patients, the mean primary tumor size was 3.39 cm, 18 patients were T1 diseases, 145 were T2, 22 were T3 and 2 were T4 (according to breast ultrasound and MRI evaluation before NACT), 145 patients showed clinical positive axillary lymph nodes (cN+). Figure 1 shows the distribution of histological subtypes (achieved by CNB) according to MG appearance before NACT. Among 187 patients with malignant calcifications, 54 had calcifications alone, 40 occurred with architectural distortion (AD) and 93 with mass in their images. The proportions of HR+HER2−, Triple Positive, HR−HER2+ and Triple Negative tumors were 43.3%, 31%, 20.3% and 5.4%, respectively, in patients with calcifications, indicating higher rates of HR positive tumors (74.3% versus 64.6%) and HER2 positive tumors (51.3% versus 33.4%), *p* = 0.004. The distribution of the histological subtypes in patients with only calcifications was similar to the distribution in those with mass or AD, *p* = 0.245. Patients with calcifications had a slightly higher pCR rate compared to those without calcifications, 35.4% versus 29.8%, but this difference was not statistically significant. Patients with calcifications only showed a statistically significantly higher rate of pCR compared to patients with mass or AD, 45.3% versus 31.3%, *p* = 0.072.

### Patterns of change in calcification before and after NACT

Two MG exams were performed for 120 patients, conducted before and after NACT. Changes were observed in range and density. Changes in range were observed for 57 patients (47.5%), with 48 becoming narrowed and only 9 broadened. The majority of patients (93/120) exhibited no change in calcification density, while 9 patients exhibited an increased density (3 patients developed new calcifications) and 18 exhibited a decreased density. Although patients with narrowed ranges exhibited a higher rate of pCR, 27.1%, and patients with increases in density showed a lower pCR rate, 11.1%, none of these changes correlated with the tumor response rate (Table 1). Very few patients exhibited morphological changes. 7 out of 120 patients who had pleomorphic calcifications changed to fine branching or casting patterns after NACT, 1 achieved tpCR, and 3 had remaining DCIS. The pCR rate of these patients was 57.1%, which was higher than the rate of those without morphological changes (33.6%, *p* = 0.044).

**Table 1:** Changes in calcifications and tumor response rates.

				Tumor Response		
		n	None	DCIS	IDC	
Range*	Narrowed	48	27.10%	12.50%	60.4	
	Broadened	9	11.1	22.2	66.7	
	Stable	63	20.6	11.1	68.3	*p* = 0.717
						
Density*	Increased	9	11.1	0	88.9	
	Decreased	18	22.2	16.7	61.1	
	Stable	93	23.7	12.9	63.4	*p* = 0.587

*Increases in the range diameter of more than 20% were defined as broadened, and reductions of more than 20% were defined as narrowed. A change in density of more than 20% was defined as a change.

### Calcifications appearances

Table 2 reports the difference between calcification appearance and pathological features. To evaluate the predict value of MG calcifications to pCR rate, we used the first MG image (before NACT) to analyzed. Calcification appearances were assessed in terms of five aspects: morphology, distribution, range, diameter, and density. The results show that 166 (91.7%) patients mainly had pleomorphic calcifications, and only 15 patients showed fine branching or casting calcifications. Diffuse or scattered calcifications were observed in 22 patients (11.8%), while grouped or clustered, segmental and regional patterns were observed in 27% of all patients. Before NACT, the mean tumor size of these patients measured by sonography was 57 mm and the mean range of calcifications was 33 mm. Calcifications with ranges greater than 2 cm were observed in 107 patients (59%), 92 patients had calcifications with diameters less than 0.5 mm, and 24 patients showed chunky calcifications with diameters greater than 1 mm. High density calcifications of >20 per cm^2^ were noted in 74 patients. No statistically significant differences were observed between the calcification appearance and the distribution of tumor subtypes. The tumor response rates were also similar between different calcification patterns. Calcifications with pleomorphic morphologies, diffuse or scattered distributions, >2 cm in range, 0.5–1 mm in diameter, and <20/cm^2^ in density exhibited relatively higher pCR rates, but none of these differences were statistically significant. Some calcification characteristics were associated with higher rates of change after NACT. Pleomorphic calcifications with a range >2 cm had higher rates of change in density. Calcifications >2 cm in range, > = 1 mm in diameter, and >20/cm^2^ in density showed higher rates of change in range, with both narrowing and broadening observed, *p*<0.05. Difference distributions also exhibited various rates of change in range.

**Table 2:** Pathological features and patterns of change in calcification for calcifications with different appearances.

		n = 181		Subtype %					Density			Range	
			HR+	HER2+	TN	ki67≥30	pCR	Increased	Decreased	Stable	Narrowed	Broadened	Stable
Morphology	Fine branching or casting	8	75	50	0	100	50	0	16.7	83.3	83.3	0	16.7
	Pleomorphic	166	74	52	6	76	36.1	7.2	13.5	79.3	36.9	8.1	55
	Combined	7	75	50	0	80	0	50	50	0	50	25	25
									*p* = 0.03			*p* = 0.124	
Distribution	Grouped or Clustered	53	74	46	9	76	28.3	2.9	5.7	91.4	20	2.9	77.1
	Linear	6	50	50	0	83	33.3	25	0	75	25	25	50
	Segmental	51	79	52	2	83	41.2	11.8	20.6	67.6	52.9	14.7	32.4
	Regional	49	73	51	8	68	28.6	10.8	18.9	70.3	48.7	8.1	43.2
	Diffuse or Scattered	22	75	63	0	85	54.5	0	18.2	81.8	36.4	0	63.6
									*p* = 0.288			*p* = 0.016	
Range (cm)	≤2	74	76	45	7	75	29.7	3.9	9.6	86.5	25	3.8	71.2
	>2	107	73	55	5	78	39.3	11.6	18.8	69.9	50.7	11.6	37.7
									*p* = 0.084			*p* = 0.001	
Diameter (mm)	≤0.5	92	74	47	7	74	32.6	9	16.4	74.6	35.8	10.5	53.7
	≤1	65	74	55	5	80	41.5	7.7	10.3	82	33.3	5.1	61.6
	>1	24	77	58	0	84	29.2	6.7	20	73.3	73.3	6.7	20
									*p* = 0.872			*p* = 0.05	
Density (per cm2)	≤20	107	71	52	7	76	39.3	7.6	15.2	77.2	29.1	8.9	62
	>20	74	80	51	3	80	29.7	9.5	14.3	76.2	59.5	7.2	33.3
									*p* = 0.931			*p* = 0.004	

### Location of the calcifications with mass

**Figure 2:**
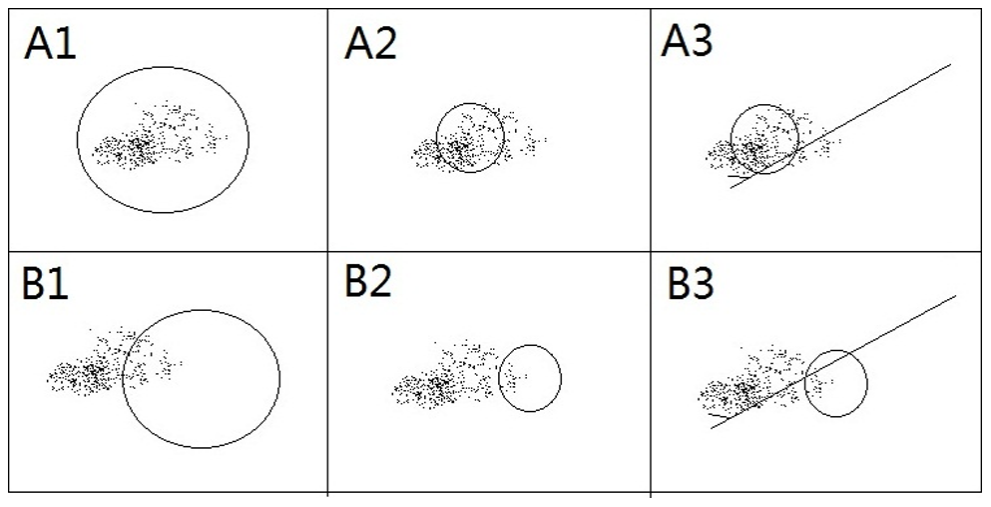
Locations and patterns of change in calcifications. A: Calcifications inside the mass; B: Calcifications outside the mass; 1: Before NACT; 2: After NACT; 3: Before operation. Circle represents mass, dots represent calcifications, and the line represents a wire.

As shown in Figure 1, 29% of patients only had malignant calcifications as assessed by MG. As it is difficult to define the range of AD by MG, only 93 patients (50% of all 187 patients) with calcifications with mass were selected to analyze whether the calcification location (inside or outside of the mass) matters. This analysis revealed that 41 (44%) patients had calcifications inside the mass, while 52 (56%) patients had calcifications exceeding the mass range, and similar tumor response rates were observed between groups, as shown in Table 3. After NACT, 9.8% of patients in the inside group and 5.8% in the outside group exhibited an increased density of calcifications. 4.8% of patients in the inside group and 5.7% in the outside group had broadened in range. No differences were observed in the patterns of change between these two groups. Figure 2 shows the locations of these calcifications and their patterns of change.

**Table 3:** Correlations between locations of calcifications and tumor response rate and patterns of change.

				Tumor Response				Density			Range	
	n	None	DCIS	IDC	pCR	tpCR	Increased	Decreased	Stable	Narrowed	Broadened	Stable
Inside	41	17	9.8	73.2	26.8	17	9.8	9.8	80.4	39.1	4.8	56.1
Outside	52	23.1	11.5	65.4	34.6	23.1	5.8	21.1	83.1	46.2	5.7	48.1

## Discussion

This study was undertaken to evaluate whether the appearance of malignant calcifications has value for prediction and surgical decision making in neoadjuvant settings. It is critical to have a sensitive and accurate method to identify tumor response and to assess the extent of residual disease after NACT to inform decisions by the surgeon and patient on the timing of surgery and the best surgical approach to minimize disease recurrence. Many previous studies have studied the correlation between the pathologically assessed residual tumor sizes and tumor sizes assessed by different examinations. Chagpar reported that physical examination, sonography and MG were only moderately useful in predicting residual pathologic tumor size after NACT [20]. Recent studies suggested that sonography [10], [18] and/or MR [19], [22] imaging were more sensitive than MG, provided better correlation with the pathological assessments of response and residual tumor size.

The infiltrating nature of breast cancer growth and the dense breast tissue are the two major factors that make size determination by MG difficult, leading to a poor predictive value of the NACT response rate. Only when combined with sonography and/or MRI can MG provide highly accurate predictions of pathological residual tumor size [10], [26]. Only a few studies have focused on the MG assessment of malignant calcifications in a neoadjuvant setting. One study reported that of 99 patients who underwent NACT, microcalcifications in MG were only observed for 10 of these patients, the number of particles increased in 2, remained stable in 3, decreased in 4, and disappeared in one patient [27]. Other studies reported that calcifications may persist, disappear or even increase in patients who respond to NACT [28]–[31].

However, these studies featured small sample sizes, analyses that were not comprehensive, and descriptions of the characteristics and change patterns of microcalcifications lacking in detail, with ambiguous clinical value for surgical decision making.

In the present study, we found that one-quarter of patients had mammographic malignant calcifications in our database. As these results are based on a prospective single-arm NACT trial, patients with large operable or locally advanced breast cancer were continuously enrolled. HER2 positive tumors are more aggressive, doctors were more willing to refer Her2 positive patients into this trial, so the patient population featured a higher proportion of HER2 positivepatients, that is, approximately 50%. The distribution of histological subtypes was significantly different between patients with and without malignant calcifications. Patients with malignant calcifications had more HR positive tumors and HER2 positive tumors, *p* = 0.004. Inconsistent with previous results indicating that responses were not complete in any of the 44 (46%) cases of microcalcifications [27], our study showed similar pCR rates in patients with or without microcalcifications. Calcifications alone were observed in 29% of the study population, while 21% occurred with architectural distortion (AD) and 50% occurred with mass. While the distributions of histological subtypes were similar among these three groups, our study demonstrated that patients with calcifications only had a higher pCR rate compared with patients with mass or AD (45.3% versus 31.3%, respectively).

In the current study, calcifications were reported by five measurements, according to their morphology, distribution, range, diameter and density. In 120 patients with two MGs, the main changes before and after chemotherapy occurred in range and density. Esserman et al reported 3 cases and demonstrated that microcalcifications can increase, decrease or remain unchanged following NACT [32]. Other studies also suggested that microcalcifications were a permanent sign of the extent of the primary lesion [33] that could decrease in number but rarely disappeared [34]. Our study showed 63 patients (52.5%) without changes in range and 93 patients (77.5%) without changes in density, and none of the changes, narrowed (n = 48) or broadened (n = 9) in range, increased (n = 9) or decreased (n = 18) in density, had statistically significant effects on the response rate (Table 1). Our results demonstrated that the persistence of calcifications did not necessarily indicate residual disease. Density increased in 9 patients, including 3 patients with newly developed microcalcifications after NACT in the primary tumor site, none of which showed progressive disease.

According to the literature, there are two types of calcification molecules in breast tissue: calcium oxalate, which is crystalline, and a non-crystalline form of calcium phosphate [35]. Two types of calcification processes take place: a secretory process that is likely to take place in benign lesions, and a necrotic process that mainly occurs in malignancies. Thus, the persistence of microcalcifications can be explained by the calcification of necrotic material remaining from the primary tumor [28].

The tumor shrinkage modes ‘concentric shrinkage’ and ‘shrinkage with residual multi-nodular lesions’ can be used to explain changes in the calcification range or density. ‘Concentric shrinkage’ may induce a decrease in the range and an increase in the density. Two pathological mechanisms may also account for these changes: multinucleated histocytes may engulf calcium deposits in patients with decreased degrees of calcification, and necrotic carcinomas can undergo calcification in patients with increased degrees of calcification [32]. However, these theories cannot explain all cases, as more than half of the patients in our study had stable calcifications. None of these patterns of change were associated with the tumor response rate.

Next, we analyzed in detail whether different microcalcification appearances were correlated with the tumor response rate and patterns of change. To the best of our knowledge, no studies have ever explored the role of microcalcification appearances in neoadjuvant settings. None of the calcification patterns exhibited any relationship to tumor histology subtypes or pathological response rates. Based on these results, we suggested that mammographically detected calcifications had no predictive value of tumor response in NACT. Considering that the patterns of change in calcification were not associated with tumor response rate either, we were skeptical that the evaluation of predicted response and residual tumor size using MG could provide a sensitive assessment of chemotherapy efficacy in NACT. Therefore, the greatest value of MG lies in its diagnostic value. We argue that only one MG exam before NACT is sufficient, and this exam is useful for diagnosis and essential for surgical decision making.

The use of BCT in patients with large breast tumors downstaged by NACT remains controversial. Some investigators reported unacceptably high local recurrence rates ranging from 10 to 20% after NACT and BCT [36]–[38], while others published acceptably low recurrence rates [39], [40]. Some surgeons are highly cautious about using BCT for patients with microcalcifications observed by MG, as the presence of calcifications on preoperative MG was associated with an increased risk of local recurrence, even though the presence of calcifications did not affect the rates of overall survival, disease-free survival, or cause-specific survival [41]. Especially for patients with calcifications located outside the mass before NACT that persisted after chemotherapy and mass down staging, surgeons and patients often choose mastectomy rather than BCT due to fear over the unclear residual tumor boundary (Figure 2). Approximately 12% of patients in our trial received BCT after NACT, while less than 5% of patients with calcifications received BCT, although many of these patients achieved completed pathological responses with the persistent presence of calcifications on their MG. As shown in Table 1, patients with no changes in range and density also had similar pCR rates, 31.7% and 36.6% respectively. Therefore, we also reported whether calcifications were present inside or outside of the tumor and studied whether this location was associated with the tumor response rate. Calcifications that occurred outside of the mass showed similar pCR rates as those inside the mass, suggesting that these patients were still eligible for breast conservation as long as there was complete excision of the persistent areas of the primary mass and calcifications with adequate margins. As shown in Figure 2B, for patients with calcifications outside of the mass after NACT when sonography and/or MRI demonstrated tumor shrinkage, a wire could be introduced before the operation to guide complete excision of the calcified area. The current study has some limitations. The measurements of microcalcifications were time-consuming and hard to be accurately, which limited the dissemination of our results. All the patients were selected from a neoadjuvant trial, among them 51.3% were Her2 positive, which may interfere the result of relation between calcifications and tumor response rates, as Her2 positive tumor had relatively higher pCR rate. However, in the same subtype patients (HR+HER2−, Triple Positive, HR−HER2+ or Triple Negative), pCR rates were similar in patients with or without microcalcifications. 61 patients missed the second MG after NACT, only two thirds of the patients (120/187) have both MG images, the relationship between changes in calcifications and tumor response rates could only be verified in this part of cases. So the first step, we analyzed whether the patterns of calcifications change significantly after NACT in those 120 patients, and found no significant changes. Then when considering to evaluate the predict value of calcification, we used 187 mammogram images before NACT to analyze the relationship between different calcification patterns with pathological features and pCR rates.

## Conclusion

This study demonstrated that patients with malignant calcifications had more HR positive tumors and Her2 positive tumors. MG may not be an accurate assessment of tumor response in NACT, various patterns of calcification appearance had similar pCR rates, calcifications did not change significantly after NACT. Persistent calcifications did not indicate residual tumors, patients with calcifications occurring outside of the mass after NACT could still be treated by BCT.
